# Effect of lidocaine on cognitively impaired rats: Anti‐inflammatory and antioxidant mechanisms in combination with CRMP2 antiphosphorylation

**DOI:** 10.1002/iid3.1040

**Published:** 2023-10-11

**Authors:** Xiaohong Zheng, Yuerong Lin, Linshen Huang, Xianzhong Lin

**Affiliations:** ^1^ Department of Anesthesiology, First Affiliated Hospital Fujian Medical University Fuzhou China; ^2^ Department of Anesthesiology, National Regional Medical Center, Binhai Campus of the First Affiliated Hospital Fujian Medical University Fuzhou China

**Keywords:** cognitive dysfunction, collapsing response mediator protein‐2 (CRMP2), inflammation, lidocaine, oxidative stress

## Abstract

**Objective:**

Studies have shown that lidocaine has antioxidative stress, anti‐inflammatory, and nerve‐protective effects. The current study investigated the effects of lidocaine on cognitive function in rats with cognitive dysfunction.

**Methods:**

A total of 48 rats were randomly assigned to four groups of 12 rats each: control group; L (lidocaine) + D (d‐galactose) group, d‐galactose group (D group); and D + L group. We assessed cognitive function using a Morris water maze (MWM) and pathologic changes of hippocampal sections. An enzyme‐linked immunosorbent assay (ELIZA) was used to detect serum malondialdehyde (MDA) and superoxide dismutase (SOD) levels in rats, and protein immunoblotting (western blot) was used to detect brain tissue proteins (collapsing response mediator protein‐2 [CRMP2], phosphorylated‐collapsing response mediator protein‐2 [P‐CRMP2], and β‐amyloid protein [Aβ]).

**Results:**

The MWM showed that the d‐gal group (284.09 ± 20.46, 5.20 ± 0.793) performed worse than the L + D (265.37 ± 22.34, 4.170 ± 0.577; *p* = .000) and D + L groups (254.72 ± 27.87, 3.750; *p* = .000) in escape latency and number of platform crossings, respectively. The L + D group (44.94 ± 2.92 pg/mL, 6.22 ± 0.50 pg/mL, and 460.02 ± 8.26 nmol/mL) and D + L group (46.88 ± 2.63 pg/mL, 5.90 ± 0.38 pg/mL, and 465.6 ± 16.07 nmol/mL) had significantly lower serum inflammatory levels of interleukin‐6, tumor necrosis factor‐α, and MDA than the d‐gal group (57.79 ± 3.96 pg/mL, 11.25 ± 1.70 pg/mL, and 564.9 ± 15.90 nmol/mL), respectively. The L + D group (3.17 ± 0.41 μg/mL) and D + L group (3.08 ± 0.09 μg/mL) had significantly higher serum inflammatory levels of SOD than the d‐gal group (2.20 ± 0.13 μg/mL) (all *p* = .000). The levels of CRMP2, P‐CRMP2, and Aβ in the brain tissue homogenates of the L + D group (0.87 ± 0.04, 0.57 ± 0.0, and 0.16 ± 0.02) and the D + L group (0.82 ± 0.05, 0.58 ± 0.09, and 0.15 ± 0.02) were significantly different than the d‐gal group (0.67 ± 0.03, 0.96 ± 0.040, and 0.29 ± 0.05).

**Conclusions:**

Lidocaine was shown to reduce cognitive impairment in rats with cognitive dysfunction through anti‐inflammatory and antioxidative stress mechanisms in combination with CRMP2 antiphosphorylation.

## Graphical abstract

From the study, lidocaine may reduce cognitive impairment in rats with cognitive dysfunction through anti‐inflammatory and antioxidative stress mechanisms in combination with collapsing response mediator protein‐2 antiphosphorylation.

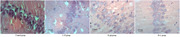

## 1 INTRODUCTION

The global population living with dementia is estimated to be as high as 47 million.^1^ Regional differences exist in the prevalence of dementia; the annual incidence of dementia is 7.5 and 8.0 per 1000 individuals worldwide and in China, respectively.^2^ Alzheimer's disease (AD) accounts for two‐thirds of dementia cases and the incidence of AD is increasing each year in parallel with the increase in the aging population in China.^2^ Mild cognitive impairment increases the likelihood that AD will develop. Once AD occurs, AD is difficult to reverse and eventually leads to decreased mobility and self‐care ability of patients, thus causing an incalculable economic burden to society and families.^3^ Early assessment, early diagnosis, and early comprehensive treatment improve the quality of life in AD patients.^3^

Recently, d‐galactose (d‐gal)‐induced aging models have been frequently used to study age‐related disease alterations and intervention effects.^4^ It has been reported that brain aging model rats had significantly reduced spatial learning and memory abilities, elevated levels of induced oxidative damage, elevated levels of inflammation, and AD‐related specific pathologic alterations, with massive deposition of β‐amyloid protein (Aβ) in brain tissues.^5^ Furthermore, synaptic impairment caused by Aβ‐induced phosphorylation of collapsing response mediator protein‐2 (CRMP2) is a critical step affecting cognitive function.^6^ The intracellular protein, CRMP2, mediates repulsive axon guidance molecules. CRMP2 is phosphorylated in the pathologic state and can be deposited in neurofibrillary tangles in the brains of AD patients.^7^ Wang et al.^8^ showed that d‐gal induces cognitive impairment and alters the expression of inflammatory factors in the mucosal intestines, which may modulate neurotransmission, thus causing the pathogenesis of cognitive impairment in reverse.

Studies have shown that lidocaine has antioxidative stress and anti‐inflammatory effects. Lidocaine induces mitochondrial depolarization through ion channels, blocks the tricarboxylic acid cycle, reduces the energy supply of oxidative damage processes, decreases apoptosis, blocks cell membrane hydrogen peroxide lipid peroxidation reactions, and reduces free radical production.^9^ Lidocaine prevents neutrophil adhesion, migration, and chemotaxis to endothelial cells and collagen through a special sodium channel based on in vitro studies.^10^ Lidocaine also has anti‐inflammatory effects by lowering TNF‐induced nuclear transcription factor expression in peripheral blood neutrophils or partially reversing TNF‐induced neutrophil apoptosis.^11^ Brain acetylcholinesterase activity is decreased following the peripheral and systemic injection of lidocaine.^12^ Intravenous lidocaine considerably speeds up the restoration of neurologic function after acute cerebral ischemia.^13^ Patients who have undergone heart surgery may benefit from increased lidocaine administration as a neuroprotective agent.^14^

Lidocaine could lessen the cognitive impairment brought on by isoflurane anesthesia by lowering mitochondrial damage. Lidocaine reduced the ratio of Bax, an apoptosis‐promoting protein, to Bcl‐2, an apoptosis‐inhibiting protein, in H4 cells.^15^ In the current study, we determined how lidocaine affects rat cognitive function and the potential underlying mechanisms.

## 2 METHODS

### 2.1 Drugs and reagents

d‐galactose (d‐gal) was purchased from Amresco. Interleukin‐6 (rat IL‐6) and rat superoxide dismutase (SOD) enzyme‐linked immunosorbent assay (ELIZA) kits were provided by Shanghai Xitang Biotechnology Co. Technology. Antibeta amyloid antibodies were purchased from Santa Cruz Biotechnology, Inc. Lidocaine was obtained from Hunan Kelun Pharmaceutical Co., Ltd. (Product lot number, F210620A).

### 2.2 Animals and treatments

Forty‐eight clean‐grade male healthy SD rats (3 months old, weighing 350 ± 30 g) were purchased from Fuzhou Minhou County Wu's Animal Co., Ltd. (Shanghai Slaughter Laboratory Animal Co., Ltd. ex works, license number: SCXK(Hu)2017‐0005; Experimental Institution Test Using license number: SYXK (Min) 2016‐0007). The EU Directive (2010/63/EU) for animal research was followed in conducting every experiment. In well‐ventilated cages with ambient temperatures between 22°C and 23°C and relative humidity levels between 25% and 30%, rats were kept in a conventional, pathogen‐free setting with a 12‐h/12‐h dark/light cycle. Food and water were available ad libitum throughout the experiment. The rats spent 1 week acclimating to the surroundings before the study. The rats in each group were cage‐fed according to the group. The weight of each rat was measured at 8 a.m. in the morning and the general condition, including hair, spirit, foraging behavior, and action ability, was observed (Tables 1 and 2).

**Table 1:** Body weight of rats (*n* = 12 in each group).

Group	Weight before modeling (g)	F	*p* Value	Weight after modeling (g)	F	*p* Value
Control	327.78 ± 5.00	1.560	.212	404.89 ± 4.13	0.873	.463
L + D	327.86 ± 6.73			407.16 ± 4.67		
d‐gal	325.68 ± 5.85			405.97 ± 4.38		
D + L	327.99 ± 5.78			404.62 ± 4.79		

Abbreviations: D, D‐galactose; L, lidocaine.

**Table 2:** Speed of rats before and after modeling and before and after drug administration (*n* = 12 in each group).

Group	Speed of rats before modeling (mm/s)	F	*p* Value	Speed of rats after predrug modeling (mm/s)	F	*p* Value	Speed of rats after modeling and drugging (mm/s)	F	*p* Value
Control	288.37 ± 9.71	1.116	.353	271.52 ± 20.46	0.703	.555	257.75 ± 33.16	2.988	.041
L + D	281.88 ± 26.24			269.88 ± 26.24			265.37 ± 22.34		
d‐gal	281.28 ± 25.55			270.99 ± 26.14			284.09 ± 20.46		
D + L	271.37 ± 26.11			282.27 ± 22.04			254.72 ± 27.87		

Abbreviations: D, D‐galactose; L, lidocaine.

### 2.3 Establishment of the cognitive dysfunction rat model and experimental groupings

A total of 48 male rats were randomly assigned to four groups of 12 rats in each group after Morris water maze (MWM) testing^16^: control group; the lidocaine + d‐galactose (L + D) group; d‐gal group (the D group served as the modeling group^17^); and the d‐galactose + lidocaine (D + L) group. The day following the MWM test, the rats in the L–D group were injected intraperitoneally with lidocaine (3 mg/kg^−^^1^), and the other three groups were injected intraperitoneally with the same dose of normal saline. The rats in the L + D, D, and D + L groups were injected subcutaneously withd‐gal (1000 mg/kg^−^^1^/D‐1) for modeling 1 week later, while the rats in the control group were injected subcutaneously with the same dose of normal saline. The MWM test (5 days) was performed in all four groups the day after the modeling was completed. The D–L group rats were injected intraperitoneally with lidocaine (3 mg/kg^−^^1^) the day after the MWM test was completed, and the remaining three groups were injected intraperitoneally with the same dose of normal saline for 1 week. Then, the MWM test (5 days) was performed the day after drug injection.

### 2.4 Evaluating cognitive function with the MWM test

The 5‐day MWM test^16^ was performed to confirm the presence of changes in cognitive function in rats. The MWM test consists of a water maze device and a water maze image automatic acquisition and software analysis system. The system automatically collects the parameters of the animal's entry position, swimming speed, time required to search the target, running track, and search strategy and can statistically analyze all of the collected data. The personnel and environment were unchanged and interference by reference objects was excluded during the entire experimental process.

**Figure 1:**
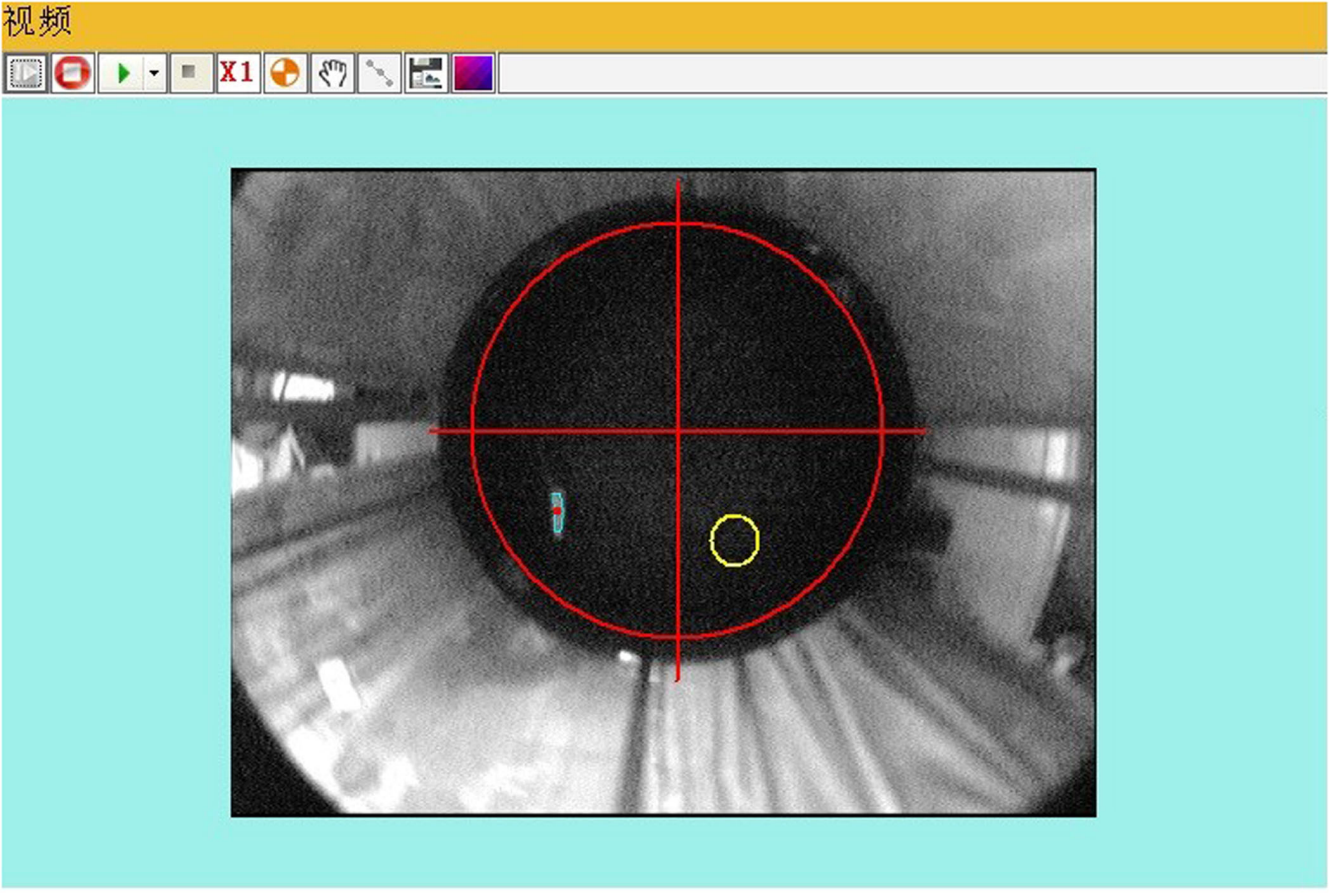
The Morris water maze test device.

The device for the MWM test (Shanghai Xinsoft Information Co., Ltd.) is shown in Figure 1. The MWM device mainly consists of a pool of water and a platform with an adjustable height and movable position. The water maze pool is cylindrical with a diameter of 1.8 m and a height of 0.5 m. The platform is generally round with a diameter of 12 cm, a rough surface, and a height of 30 cm.

The water temperature remained constant during the experiment. The temperature range of rats is generally 25 ± 1°C. There was no light or shadows on the water surface of the pool. A black curtain was installed around the pool to reduce the outside people standing somewhere next to the pool or walking around, thus forming an indefinite space reference for rats.

The main components of the water maze image automatic acquisition and software analysis system were a camera, computer, and image acquisition card. The camera captured the rat swimming image (analog signal) and input the image into the image acquisition card of the computer for analog/digital conversion. The analog image of the rat swimming was converted into a digital image and stored in a hard disk, and the digital image was analyzed to achieve the relevant test parameters. The software analysis system automatically collected the parameters of the animal's entry position, swimming speed, time required to search the target, running track, and search strategy and statistically analyzed all of the collected data.

The MWM test mainly assesses spatial learning and memory. The main experiments include hidden station and space exploration experiments.

The concealed platform test was performed at the beginning of each day. Rats were placed into the water from any of the four quadrants (northeast, northwest, southeast, and southwest) facing the pond wall, and the platform was placed in the southeast quadrant. Each time, the experimental rats swam a total of 90 s to find the hidden platform. If the rat successfully found the platform, the rats were given 10 s of rest on the platform. If the platform is not found successfully in 90 s, the rats are manually placed on the platform, and given 10 s of rest. Sometimes, the rat fell off the platform or jumped into the water to continue swimming before the 10–30 s interval expired. Once this occurred, the rat was put back on the platform and the 10–30 s time interval was restarted. In so doing, each rat had an equal amount of time to observe and acquire spatial information after each experiment. Each of the four quadrants were completed in turn, and the interval between each experiment was 10–15 min for 5 days. During the entire experimental process, the personnel and experimental environment was unchanged and the reference object interference was excluded. With the use of special software, the computer synchronously tracked and recorded the track, time, distance, and swimming speed of the rats looking for the platform to obtain the relevant result escape latency data.

Twenty‐four hours after completion of the concealed platform test, the platform was removed. Then, the rats were put into the water according to the original entry sequence, and the swimming path of the rats within 60 s was recorded, as well as the residence time of the rats in the original platform quadrants and the number of times the rats crossed the original platform. The spatial positioning ability of the tested rats was observed during the spatial exploration test and the change rule in the process of space exploration was observed. The original location of the platform was marked with a circular ring on the computer screen so that the number of times at the original location of the platform was recorded. The collected data represented platform crossing.

### 2.5 ELIZA was used for determination of serum IL‐6, TNF‐α, SOD, and malondialdehyde (MDA) in rats

The rats were starved for 12 h following the last MWM test, then six rats were randomly chosen from each group. All rats were anesthetized with 250 mg/kg^−1^ of 10% chloral hydrate (hospital prepared) intraperitoneally. After 1–2 min rat resistance weakened, as evidenced by natural drooping of the limbs and tail, but respirations were unchanged. The rats were placed on a bench in the supine position and immobilized. Blood was drawn from the blood vessel from tail veil, and between 3 and 5 mL of blood was centrifuged at 3000 rpm for 8 min. The supernatant was stored in a freezer at −4°C. The chest and abdomen were then opened quickly to expose the heart and liver. The heart was gently lifted with the tweezers in the left hand, a trocar was inserted into the apex of the heart with the right hand, and the needle was directed up to the ascending aorta. The inner core of the trocar was removed, connected to a normal saline solution, the infusion switch was turned on, and the rat was quickly perfused. A small opening was made in the right auricle with scissors and normal saline was replaced with polyformaldehyde when the color of the outflow liquid turned pale and the liver turned gray. The perfusion rate of paraformaldehyde was initially fast, then slow. After the rapid perfusion of 50 mL, the perfusion rate was slowed. The perfusion rate was maintained until the limbs and muscles of the entire body spontaneously twitched and the tail was straight. When the involuntary twitching stopped and the organs of the entire body were stiff, the head was severed by a guillotine and the skin was removed with tissue scissors. The skin was cut in the middle of the skull to expose the skull. Ophthalmic scissors were inserted into the vertebral foramina from the spinal cord end and the skull was cut along the median line. Care was taken to reduce brain tissue damage at points where the skull adhered to the scissors. After cutting the skull, a vascular clamp was used to separate the skull from the brain until the entire brain was exposed. The entire brain was removed and was then soaked in 4% paraformaldehyde fixing solution for 24 h. The next day, paraffin embedding was performed to determine the anatomic location of the hippocampus, and continuous coronal sections were cut and hematoxylin and eosin (HE) staining^18^ was performed. The sections used for histologic evaluation were 4‐um thick (Leica RM2245).

The IL‐6 content (Shanghai Xitang Biotechnology Co. Technology), tumor necrosis factor‐α (TNF‐α) (Shanghai Xitang Biotechnology Co. Technology), SOD (Shanghai Xitang Biotechnology Co. Technology), and MDA (Shanghai Xitang Biotechnology Co. Technology) in serum were detected in the collected blood. The pathologic morphologic changes of hippocampus tissues were observed by HE staining under 4×, 10×, and 40× using a Ci‐L upright clinical microscope (Nikon).

### 2.6 Detection of brain proteins (CRMP2, phosphorylated‐collapsing response mediator protein‐2 [P‐CRMP2], and Aβ) by western blot analysis

CRMP2 (CST), P‐CRMP2 (CST), and Aβ levels in the brain tissue samples were measured. The rat was immediately decapitated with tissue scissors, the scalp was cut open to expose the skull, and the skull was removed with biting forceps to expose the brain tissue. The brain tissue was removed and placed in iced saline to remove the blood. The brain tissue was then swabbed dry with filter paper, weighed, placed in a 5‐mL beaker, placed in an ice box, cut into small pieces with ophthalmic scissors, poured into a glass homogenization tube, and 4% phosphate‐buffered saline (pH 7.4) homogenization medium was added. The homogenization medium‐to‐tissue weight ratio was 1:9. The homogenate was centrifuged at 2500 rpm for 20 min to remove any remaining visible tissue fragments from the glass homogenization tube. The supernatant was then extracted using a pipette, the precipitate was discarded, and the supernatant was stored at −20°C for later freeze‐thawing.

### 2.7 Observation of histopathologic morphology changes in the hippocampus of rats

The remaining six rats in each group were anesthetized with 10% chloral hydrate (250 mg/kg^−1^) intraperitoneally after a 12‐h fast. The rats were placed on the experimental table in the supine position with the limbs fixed and the thoracoabdominal cavity was opened to expose the heart and liver. The heart was gently lifted with forceps in the left hand and a trocar needle was inserted through the apical region in the right paw and advanced upward to the ascending aorta. The inner core of the trocar needle was removed, saline was connected, the infusion switch was turned on, and rapid perfusion was initiated. Scissors were used to make a small incision in the right heart auricle, which was replaced with paraformaldehyde after the outflow fluid turned light in color and the liver became gray in color. Paraformaldehyde was infused at a rapid rate for the first 50 mL, then at a gradual maintenance rate until the muscles twitched uncontrollably and the tail stiffened. The organs and tissues were stiff and the involuntary twitching had stopped when the skull was severed by guillotine and the skin was sliced using tissue scissors to reveal the skull. Eyelid scissors were used to cut the skull along the median line after being placed into the vertebral foramen from the spinal cord end. The skull was sliced and divided with vascular forceps, taking care to avoid damaging the brain tissue until the entire brain was exposed. The nerves were then severed from the skull base and the entire brain was removed. The brain was then immersed for 24 h in 4% paraformaldehyde fixative. The following day, the brain was embedded in paraffin. Serial sections were obtained in the coronal position and stained with HE once the anatomic location of the hippocampus tissue had been established. Under a 4×, 10×, and 40× power microscope, the pathologic alterations in the hippocampus tissue were noted.

## 3 STATISTICAL ANALYSIS

SPSS 25. 0 statistical software was used for statistical analysis. The measurement data are shown as the mean ± standard deviation. One‐way analysis of variance (ANOVA) was used to compare body weights and swimming speed of rats before and after modeling. One‐way ANOVA and Tukey's post hoc test were used to compare the number of platform crossings, serologic inflammatory factors, SOD, MDA, CRMP2, and P‐CRMP2 levels, and the Aβ target band ratios in each group. Comparisons of latency to escape results before and after modeling and before and after drug administration were performed using repeated‐measures ANOVA and Tukey's post hoc test. The total sample size was estimated to be 48, with 12 samples in each group. The sample size of this study meets the statistical requirements. A *p* < .05 was considered statistically significant.

## 4 RESULTS

### 4.1 General condition

All of the rats included in the study had sound mental health and typical daily activities. There was no variation in body weights or swimming speed between before and after modeling in the four groups of rats (Table 3; *p* > .05).

**Table 3:** Body weights and swimming speed before and after modeling among all groups.

	Control group		L + D group		d‐gal group		D + L group		F		*p* Value	
	Before modeling	After modeling	Before modeling	After modeling	Before modeling	After modeling	Before modeling	After modeling	Before modeling	After modeling	Before modeling	After modeling
Weight (g)	327.78 ± 5.00	327.86 ± 6.73	327.86 ± 6.73	407.16 ± 4.67	325.68 ± 5.85	405.97 ± 4.38	327.99 ± 5.78	404.62 ± 4.79	1.560	0.873	.212	.463
Swimming speed (mm/s)	288.37 ± 9.71	257.75 ± 33.16	281.88 ± 26.24	265.37 ± 22.34	281.28 ± 25.55	284.09 ± 20.46	271.37 ± 26.11	254.72 ± 27.87	1.116	0.703	.353	.555

Abbreviations: D, D‐galactose; L, lidocaine.

### 4.2 MWM analysis

**Figure 2:**
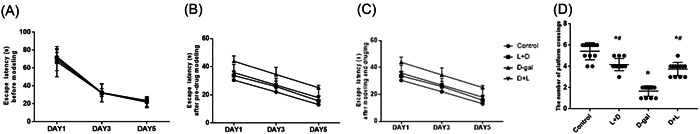
Morris water maze analysis of the escape latencies and the number of platform crossings. *Escape latencies*. (A) No statistically significant difference was detected among the groups after latency avoidance preadministration modeling. (B and C) The d‐gal group performed worse than the L (lidocaine) + D (D‐galactose) and D + L groups in escape latency (*p* < .001). *The number of platform crossings*. (D) After postmodeling administration in the spatial exploration experiment, the number of crossings between the L + D, D + L, and d‐gal groups was statistically significant.

After latency avoidance preadministration modeling, the difference was not statistically significant when compared to the d‐gal group (Figure 2A). MWM showed that the d‐gal group (284.09 ± 20.46) performed worse than the L + D (265.37 ± 22.34) and D + L groups (254.72 ± 27.87; *p* < .001) in escape latency (Figure 2B,C). After postmodeling administration in the spatial exploration experiment, the number of crossings (Figure 2D) between the L + D (4.170 ± 0.577; *p* < .001), D + L (3.750 ± 0. 662; *p* < .001), and d‐gal groups (5. 20 ± 0.793) was statistically significant.

### 4.3 Detection of IL‐6, TNF‐α, MDA, and SOD levels

**Figure 3:**
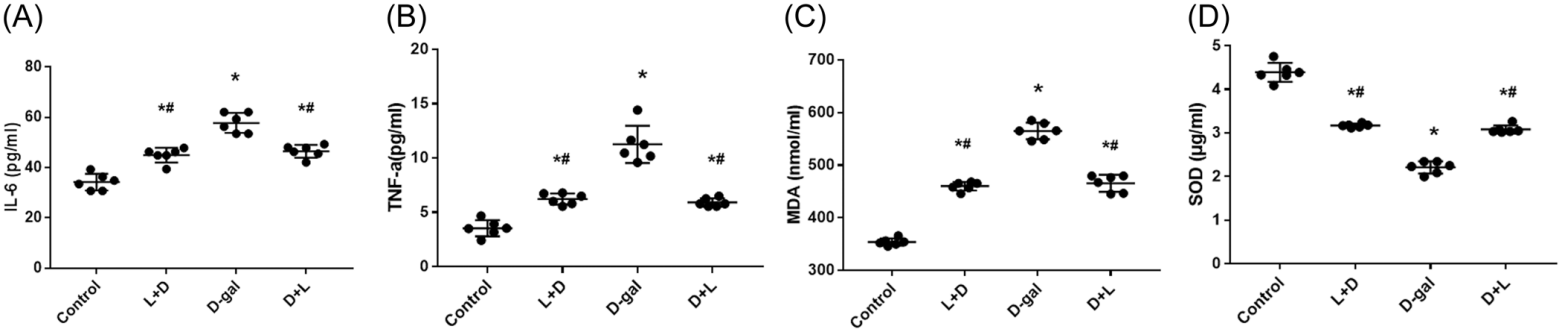
Levels of inflammatory factors and oxidative stress among the groups. (A) Interleukin‐6（pg/mL); (B) TNF‐α (pg/mL); (C) malondialdehyde (nmol/mL); (D) superoxide dismutase (µg/mL) (all *p* < .001). After postmodeling administration in the spatial exploration experiment, the levels of inflammatory factors and oxidative stress between the L (lidocaine) + D (D‐galactose), D + L, and d‐gal groups were statistically significant.

Figure 3A–D displays the serum levels of inflammatory factors and oxidative stress in the L + D group. The L + D (44.94 ± 2.92 pg/mL, 6.22 ± 0.50 pg/mL, and 460.02 ± 8.26 nmol/mL) and D + L groups (46.88 ± 2.63 pg/mL, 5.90 ± 0.38 pg/mL, and 465.6 ± 16.07 nmol/mL) had significantly lower serum inflammatory levels of IL‐6, TNF‐α, and MDA, respectively, than the d‐gal group (57.79 ± 3.96 pg/mL, 11.25 ± 1.70 pg/mL, and 564.9 ± 15.90 nmol/mL). The L + D (3.17 ± 0.41 μg/mL) and D + L groups (3.08 ± 0.09 μg/mL) had significantly higher serum inflammatory levels of SOD than the d‐gal group (2.20 ± 0.13 μg/mL; all *p* < .001).

### 4.4 CRMP2, P‐CRMP2, and Aβ levels in brain tissues

**Figure 4:**
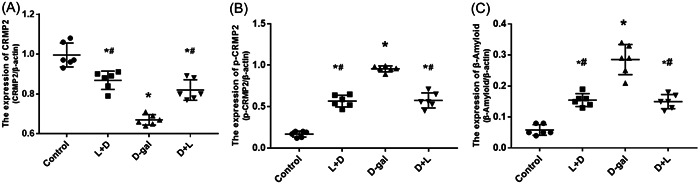
CRMP2, P‐CRMP2, and Aβ levels in the brain tissue by hematoxylin and eosin staining. (A) CRMP2; (B) P‐CRMP2; (C) Aβ, d‐gal group (0.29 ± 0.05) versus control group (0.06 ± 0.02; *p* = .000); d‐gal group, (0.29 ± 0.05) versus L (lidocaine) + D (D‐galactose) group (0.16 ± 0.02; *p* = .000); d‐gal group (0.29 ± 0.05) versus D + L group (0.15 ± 0.02) (all *p* < .001). After postmodeling administration in the spatial exploration experiment, the CRMP2, P‐CRMP2, and Aβ levels in the brain tissue between the L + D, D + L, and the d‐gal groups were statistically significant.

**Figure 5:**
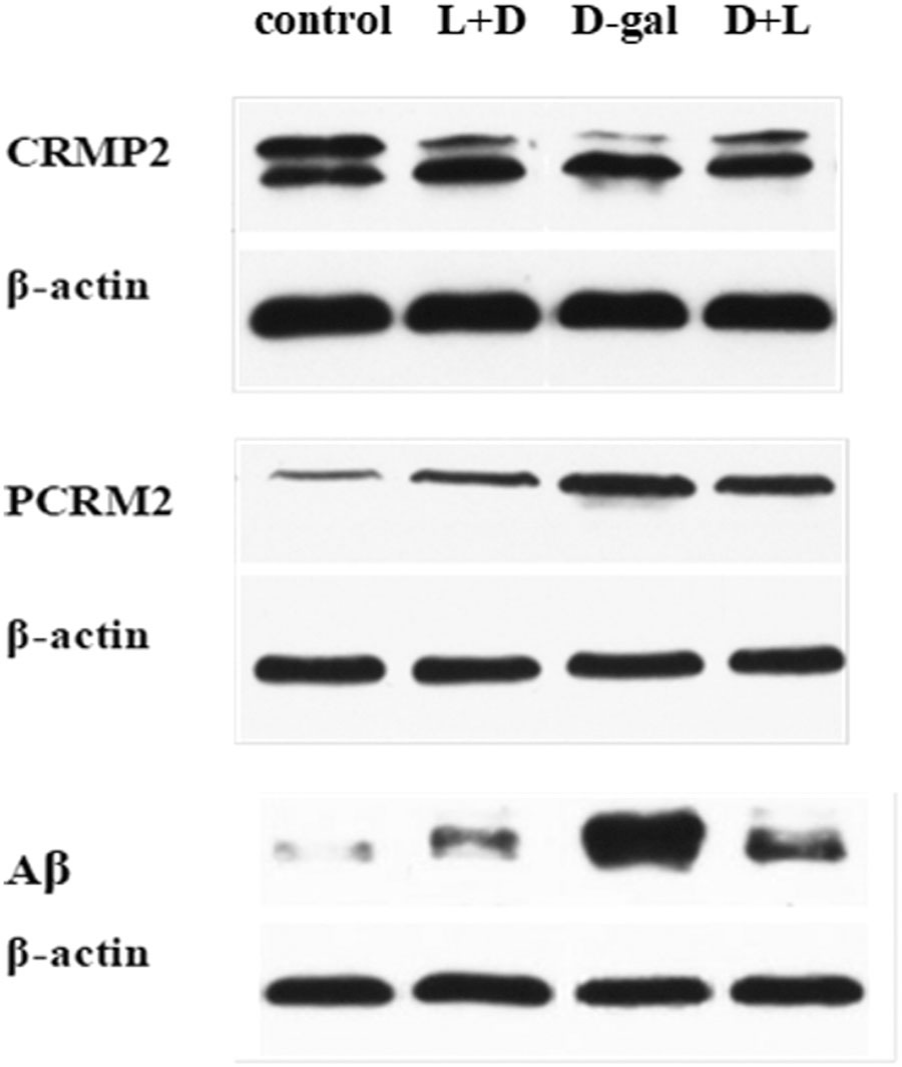
CRMP2, P‐CRMP2, and Aβ levels in the brain tissue by hematoxylin and eosin staining. (A) CRMP2; (B) P‐CRMP2; (C) Aβ, d‐gal group (0.29 ± 0.05) versus control group (0.06 ± 0.02; *p* = .000); d‐gal group, (0.29 ± 0.05) versus L (lidocaine) + D (D‐galactose) group (0.16 ± 0.02; *p* = .000); d‐gal group (0.29 ± 0.05) versus D + L group (0.15 ± 0.02) (all *p* < .001). After postmodeling administration in the spatial exploration experiment, the CRMP2, P‐CRMP2, and Aβ levels in the brain tissue between the L + D, D + L, and the d‐gal groups were statistically significant.

The CRMP2, P‐CRMP2, and Aβ levels in the brain tissue homogenates of the L + D (0.87 ± 0.04, 0.57 ± 0.0, and 0.16 ± 0.02) and the D + L groups (0.82 ± 0.05, 0.58 ± 0.09, and 0.15 ± 0.02) were significantly different from the d‐gal group (0.67 ± 0.03, 0.96 ± 0.040, and 0.29 ± 0.05; all *p* < .001, Figure 4A–C), respectively. Compared with the d‐gal group, the brain homogenates of the L + D and D + L groups had higher levels of CRMP2 and lower levels of P‐CRMP2 and Aβ, all of which were statistically different (Figure 5).

### 4.5 HE staining information

**Figure 6:**
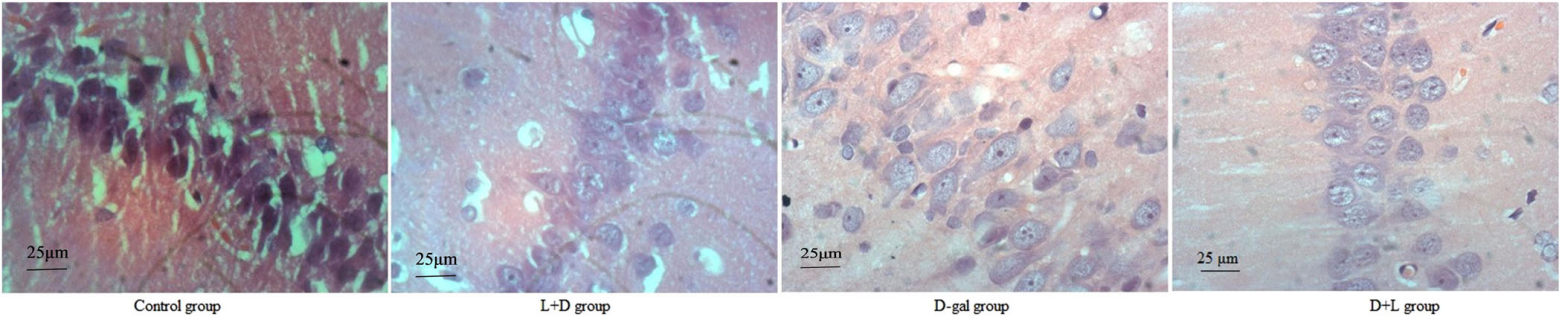
Hematoxylin and eosin staining showing the pyramidal cells in the CA1 region of the hippocampus. Bar = 25 μm. Pyramidal cells in the CA1 region of the hippocampus by hematoxylin and eosin staining at 40×. Bar = 25 μm. In the control group, the cells exhibited normal density and structure, with lightly stained, large, and round nuclei. The d‐gal group showed apoptosis of the pyramidal cells, disordered cell morphology, and significantly decreased cell number. Less apoptosis and better hippocampal cell morphology were apparent in the L (lidocaine) + D (D‐galactose) and D + L groups.

The cells in the control group had normal density and shape, as well as large, round, lightly colored nuclei. Pyramidal cells in the d‐gal group exhibited clear signs of apoptosis, disrupted cell shape, and considerably fewer cells overall. Better hippocampus cell morphology and less apoptosis were noted in the L + D and D + L groups (Figure 6).

## 5 DISCUSSION

The state of amnestic mild cognitive impairment (aMCI) is an intermediate transition stage between healthy aging and dementia and associated with a high risk of developing AD with a poor prognosis.^19^ Research has shown that the process and extent of disruption of redox homeostasis in the d‐gal aging model and in natural aging rats are similar.^20^ In the current study, the modeling was performed by subcutaneous injection of d‐gal (dose, 1000 mg/kg/d × 7 days) into the dorsal neck of rats.^17^ Mild neuropathologic alterations occurred in rat brain tissue, producing oxidative damage and leading to inflammatory activation, which is consistent with the key early mechanisms underlying aMCI disease oxidative stress and neuroinflammation.^21^

This experiment used behavioral tests (the MWM test) to confirm the presence of changes in cognitive function in rats. The MWM test is now recognized as a valid test to determine the spatial learning memory ability of aged rats. Morris first established the MWM in rats in 1981, and it was soon used as a behavioral model for research on rodent learning and memory. Hippocampal (HIP) lesions affect performance during acquisition in concealed platform training trials and subsequent probe trial performance, which has been demonstrated to be heavily dependent on HIP function for MWM task performance.^22^ Behavioral testing showed that the rats in the d‐gal group had a longer escape latency compared to the control group, and the number of platform crossings in this group was also reduced compared with the control group, indicating poor performance in the spatial exploration experiment. This finding indicated that d‐gal induced changes in cognitive function in rats, especially in memory learning ability.

The ELIZA results showed that serum MDA was increased and SOD was decreased in the d‐gal rats compared with the control rats, indicating that the level of lipid peroxidation damage in d‐gal rats was increased, and the antioxidant level of active enzymes was decreased, which further indicated that d‐gal damaged cognitive function in rats through oxidative stress. The present experiment showed that the serum inflammation level was elevated in all d‐gal group rats, indicating the existence of objective pathologic changes of neuroinflammation in the rats. SOD is an example of an antioxidant enzyme, and MDA is useful as a marker for oxidative stress. MDA has been shown to be elevated in depressed patients.^23^ Metalloenzymes comprise the SOD group. One becomes more susceptible to oxidative stress‐related disorders with aging because the enzyme has anti‐inflammatory properties and can stop precancerous cell alterations.^24^ As we age, our body's natural SOD levels decline.^25^ Observation of HIP histopathologic sections also showed that the HIP CA1 region of rats in the d‐gal group with cognitive dysfunction showed pathologic changes, such as a significant decrease in the number of pyramidal cells, disorganized arrangement, and blurred structure. These changes are similar to the pathologic changes in the HIP subregions in Parkinson's disease patients with normal aging.^26^

Related studies have confirmed that lidocaine has cerebral protective effects. Low‐dose intraperitoneal administration of lidocaine to rats significantly counteracts the toxic inflammatory changes in HIPl tissue following isoflurane anesthesia, thereby alleviating the progression of cognitive dysfunction.^27^ Lidocaine has a 1–2 h pharmacokinetic duration of action. In this experiment, lidocaine (3 mg/kg/day) was injected intraperitoneally after modeling, and the latency of evasion and the number of stage penetration were significantly improved in the L + D and D + L groups compared with the d‐gal group. The ELIZA analysis revealed that the level of inflammation in the serum of the lidocaine‐administered rats (L + D and D + L groups) was significantly reduced. In HIP pathologic histology sections, the number of pyramidal cells in the CA1 region of the hippocampus was denser and the structure was clearer in the L + D and D + L groups compared with the d‐gal group, and the degree of neuronal damage was less. All results indicate that lidocaine exerts a protective effect on the brain. According to the mechanism of action related to lidocaine studied in national and international literature, the application of lidocaine after a brain injury reduces the number of activated glial cells and decreases the expression of inflammatory factors by blocking certain sites of action on the cell surface to exert a cerebral protective effect.^28^ During treatment, lidocaine reduces posttraumatic brain injury by mechanisms related to its free radical scavenging effect or by alleviating cortical hypoperfusion injury in the early posttraumatic period.^29^ Lidocaine also alleviates apoptosis by inhibiting the mitochondrial electron transport chain and membrane, which potentially weakens and increases key enzyme activity in a dose‐ and time‐dependent manner.^30^ Feng et al.^31^ injected lidocaine (2.5, 5, 10, 20, and 40 mg/kg) intraperitoneally, and after 3 days, it was found that the death rate of animals was increased at the 20 and 40 mg/kg doses. The apoptosis of ischemic brain cells was aggravated and the neurodeficit score was low, while 2.5, 5, and 10 mg/kg had a dose‐dependent improvement in protection with dose reduction. The neurodeficit score and apoptosis were improved. We suggest that high‐dose lidocaine preconditioning has no protective effect on the brain and high‐dose lidocaine aggravates ischemia and may be neurocytotoxic, which promotes apoptosis.^31^ According to another study, intraperitoneal lidocaine doses vary from 1000 to 1 mg, and clinical toxicity rarely occurs.^32^ According to previous reports,^31^, ^32^ we considered the safety of the animals and used the 3 mg/kg dose for lidocaine in this study.

Many studies have identified the inflammatory response as the central pathologic mechanism for Aβ production and deposition. Studies have also shown that Aβ stimulates microglia to produce initiation factors as the catalyzing link in the inflammatory response. The initiation factor enhances the proliferation and activation of microglia, while promoting the release of cytokines, such as IL‐6, and inducing an increase in the content of complement, chemokines, and adhesion molecules.^33^ These immune‐inflammatory cytokines activate microglia positively, which eventually leads to neuronal cell degeneration and necrosis, senile plaque formation, and Aβ deposition. These cytokines in turn further stimulate microglia to synthesize and secrete cytokines and continue to activate microglia cells, producing a cascade amplification effect of the inflammatory response, which leads to severe neurologic damage in the brain and various degrees of cognitive impairment.^34^

Mouse models of AD have increased P‐CRMP2 levels, which is specifically present in AD, occurs downstream of Aβ production, and may also serve as a specific pathologic feature of AD.^33^ Therefore, in this experiment, we determined CRMP2 and P‐CRMP2 levels in rat brain tissue with the western blot method to elaborate the molecular objective pathologic alterations of brain injury‐related proteins in d‐gal rats. The brain‐protective protein CRMP2 was significantly decreased in the d‐gal group, while the pathologic protein, P‐CRMP2 was significantly increased.

Previous studies have shown that P‐CRMP2 interferes with neuronal assembly and CRMP2 activity is regulated by signaling‐induced phosphorylation of the C‐terminal tail region. Nonphosphorylated CRMP2 induces the formation of normal neuronal axonal microtubule proteins that serve as axonal features of neurons, whereas phosphorylated CRMP2 fails to assemble into heterotrimeric structures with microtubule proteins, resulting in blocked formation of normal axonal microtubules, collapsed growth cones, stalled neuronal axonal developmental processes, and neuronal dysfunction.^35^

To promote axonal extension, nonphosphorylated CRMP2 binds tubulin dimers; phosphorylation abolishes this action. Instead, P‐CRMP2 functions as a modulator of intracellular signaling that prevents axonal guidance, such as that mediated by semaphorin‐3A (Sema3A).^36^ Cross‐talk between oxidation and phosphorylation in Sema3A‐signaling is indicated by the CRMP2‐thioredoxin complex facilitation of CRMP2 phosphorylation at Thr509 by GSK3.^37^ CRMP3 controls HIP neuron dendritic growth, while CRMP2 is mostly expressed in axons in primary cultured cortical neurons.^38^ The diverse phenotypes produced by the gene knockout of the CRMP family proteins demonstrate the varied roles played by each member in the family. CRMPs are promising target molecules for therapeutic intervention because studies on the pharmacologic effects of CRMP‐related drugs and the phenotypic analysis of a CRMP2‐knockin mouse model with a nonphosphorylated form suggest that the phosphorylation/dephosphorylation process plays a critical role in the pathophysiology of neuronal development, regeneration, and neurodegenerative disorders.^36^ In vertebrate hippocampus neurons, lamellopodia development, neuritogenesis, and dendritic arborization are significantly influenced by CRMP3.^39^ CRMP3−/− knockout mice^40^ exhibit aberrant functional and structural neuronal networks linked to a postponement of neurite outgrowth and changes in dendrites and spines, but not axon shape in developing HIP neurons that last into adulthood. A portion of the hippocampus circuitry and function are affected by these changes. CRMP−/− mice exhibit aberrant long‐term potentiation (LTP) and a defect in prepulse inhibition, both of which are present in a number of mental illnesses. According to Quach et al.,^40^ the HIP‐fibroblast growth factor (FGF) pathways refer to the neuronal projections from HIP that either connect to the FGF directly (monosynaptic) or indirectly (polysynaptic). The anterior cingulated regions of the prefrontal cortexcam (PFC) in rats are reached through the direct monosynaptic HIP‐PFC pathway via the fimbria/fornix system, which originates from the CA1 and the subiculum. The study by Quach et al. demonstrates synaptic plasticity that is activity‐dependent, such as depotentiation or LTP/long‐term depression. Lidocaine therapy impairs the functionality of hippocampus.^40^

Our research revealed that P‐CRMP2 levels were lower in the L + D and D + L groups brain tissue compared to the d‐gal group, while CRMP2 levels were greater, indicating that lidocaine may have a role in preventing CRMP2 from phosphorylation.

This study had several limitations: This study did not dynamically monitor the specific vital signs of rats, and the study involved exposure to chronic stresses, such as injected drugs and capturing of the rats during the experiment for which the effect on cognitive dysfunction is still unknown. The use of the MWM experimental procedure also had multiple influencing factors, which brought challenges when conducting the experiment and elaboration of the results.

## 6 CONCLUSION

In summary, our analysis showed that the administration of lidocaine may improve cognitive dysfunction by reducing the phosphorylation of CRMP2 through anti‐inflammatory and antioxidant stress. The exact mechanism needs to be further investigated.

## AUTHOR CONTRIBUTIONS

Xiaohong Zheng conceived and designed the experiments, participated in the experiments, collected the clinical data, validated the data, and drafted the manuscript. Yuerong Lin and Linshen Huang participated in performing the experiments, collected the clinical data, performed the statistical analysis, and critically revised the manuscript. Xianzhong Lin collected the clinical data, interpreted the data, and reanalyzed the clinical and statistical data, and did critical revisions and editing on the manuscript. All authors gave final approval of the version to be published and agreement to be accountable for all aspects of the work in ensuring that questions related to the accuracy or integrity of any part of the work are appropriately investigated and resolved.

## ETHICS STATETMENT

All experimental procedures were conducted in accordance with the relevant ethical regulations of the Animal **Care** Committee of the First Affiliated Hospital of Fujian Medical University **(approval number, IACUC FJMU 2022‐NSFC‐0249)**. All experimental methodologies and animal housing complied with the general recommendations and provisions of the Chinese Experimental Animal Administration Legislation.

## Data Availability

The data set presented in the study is available on request from the corresponding author during submission or after publication.
